# Metropolitan Asylums Board Contracts

**Published:** 1918-10-12

**Authors:** 


### Metropolitan Asylums Board Contracts.

The Contract Committee of the Metropolitan Asylums Board has accepted the following tenders, which are of interest as an index of the conditions and prices prevailing at the present time :—

Cocoa, Prepared.—F. J. Rogers and Co., 21 Mincing Lane, E.C. 3.

Disinfectants (12 months).—The "Sanitas" Co., Ltd., Locksley Street, Limehouse, E.; (6 months) Pearson's Antiseptic Co., Ltd., 15 Elm Street, W.C. 1.

Dressings and Bandages (6 months).—S. Maw, Son and Sons, Ltd., 7-12 Aldersgate Street, E.C. 1; Sangers, 258 Euston Road, N.W. 1; May, Roberts and Co., Ltd., 7-13 Clerkenwell Road, E.C. 1; W. G. Taylor, Charford Mills, Saltley, Birmingham; A. Berton, Ltd., 15-17 Worship Street, E.C. 2; B. Pratt and Co., 57 Basinghall Street, E.C. 2.

Drugs.—British Drug Houses, Ltd., 22-30 Graham Street, City Road, N.

Druggists' Sundries (6 months).—May, Roberts and Co., Ltd.; (12 months) Leslies, Ltd., 62 London Wall, E.C. 2.

General Groceries.—G. T. Cox and Sons, Ltd., 31 King William Street, E.C.; Oliver, Oliver and Co., 231 Southgate Road, N. 1.; White, Cottell and Co., Warner Road, S.E. 5.

Ice.—United Carlo Gatti, Stevenson and Slaters, Ltd., 62-63 Queen Street, E.C. 4.

Lubricating Oils (6 months).—Prices' Co., Ltd., Battersea, S.W. 11.

Methylated Spirit.—Waters and Co., Ltd., 7-10 Bateman's Row, E.C. 2.

Surgical Appliances (12 months).—S. Maw, Son and Sons, Ltd., 7-12 Aldersgate Street, E.C.; W. H. Bailey and Son, 38 Oxford Street, W.

Soap.—James Kay and Sons, Ramsbottom; Wm. Gossage and Sons, Ltd., Widnes; Rubber Tar, Ltd., Huntershill Quarry, Bishopbriggs, near Glasgow.

Window Glass.—Pilkington Bros., Ltd., Glass Works, St. Helens, Lancs.

Cotton Hose (bulk supply).—D. Payne and Son, Ltd., Hinckley.

The tenders (each under £100) of the following firms have also been accepted: MacFarlane, Lang and Co., Fulham, S.W., biscuits; Verinder and Son, Ltd., 12 Great Eastern Street, E.C. 2, brushmakers' materials; J. Griffin, 270 Tabard Street, S.E., brushmakers' materials; R. G. Norman and Sons, Queen's Road, Watford, pig meal; R. and H. Strickland, Dartford, cattle cake, etc.; G. T. Cox and Sons, Ltd., King William Street, E.C., split peas; Civil Service Supply Association, Queen Victoria Street, E.C., currants and raisins; G. T. Cox and Sons, Ltd., rice; Price's Patent Candle Co., Battersea, S.W. 11, soap; Storr and Co., Canal Wharf, Maidstone, superphosphates; and G. T. Cox and Sons, Ltd., oatmeal.

